# Conception of a Smart Artificial Retina Based on a Dual‐Mode Organic Sensing Inverter

**DOI:** 10.1002/advs.202100742

**Published:** 2021-06-06

**Authors:** Chih‐Chien Hung, Yun‐Chi Chiang, Yan‐Cheng Lin, Yu‐Cheng Chiu, Wen‐Chang Chen

**Affiliations:** ^1^ Department of Chemical Engineering National Taiwan University Taipei 10617 Taiwan; ^2^ Advanced Research Center for Green Materials Science and Technology National Taiwan University Taipei 10617 Taiwan; ^3^ Department of Chemical Engineering National Taiwan University of Science and Technology Taipei 10607 Taiwan

**Keywords:** inverter, photodetector, photorecorder, smart artificial retina

## Abstract

The human visual system enables perceiving, learning, remembering, and recognizing elementary visual information (light, colors, and images), which has inspired the development of biomimicry visual system‐based electronic devices. Photosensing and synaptic devices are integrated into these systems to realize elementary information storage and recognition to imitate image processing. However, the severe restrictions of the monotonic light response and complicated circuitry design remain challenges for the development of artificial visual devices. Here, the concept of a smart artificial retina based on an organic optical sensing inverter device that can be operated as a multiwavelength photodetector and recorder is reported first. The device exhibits a light‐triggered broadband (red/green/blue) response, a low energy consumption as low as ±5 V, and an ultrafast response speed (<300 ms). Moreover, the multifunctional component is also combined within a single cell for health monitoring of the artificial retina during light surveillance to avoid retinopathy. Proof‐of‐concept devices, by simplifying the circuitry and providing dual‐mode functions, can contribute significantly to the development of bionics design and broaden the horizon for smart artificial retinas in the human visual system.

## Graphical abstract

An organic optical sensing inverter based on the N and P‐type field‐effect transistors is first proposed, presenting dual‐mode functions for red/green/blue photoreceptors and blue‐ray surveillance within a single cell. Through the bilayer configurations, the device exhibits a light‐triggered broadband response, low energy consumption (±5 V), and an ultrafast response (<300 ms).

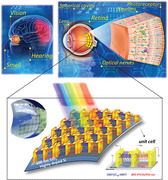

## 1 Introduction

The human visual system enables the recognition of various light, colors, and images in a complicated environment, which has inspired the development of biomimicry visual systems through electronic devices for future artificial vision.^[^ ^1^, ^2^, ^3^, ^4^ ^]^ An artificial retina is an intelligent apparatus designed as a replacement for sight in a visual system to perform the simple task of image processing of visual details. Retinal photoreceptor cells distinguish photons of different wavelengths and convert them into electronic signals for interpretation.^[^ ^5^ ^]^ For an artificial visual system, the sensory neurons in the retina can not only detect the light trigger but also perform first‐stage image processing prior to the more complex visual signal processing in the visual cortex of the human brain.^[^ ^6^, ^7^, ^8^ ^]^ However, an implanted artificial retina with profound visual loss from retinitis pigmentosa only achieved a visual resolution of 60 pixels and requires 2–3 s to recognize an object.^[^ ^9^, ^10^ ^]^ Therefore, instantaneous and accurate image recognition based on electronic devices must overcome the following challenges when targeting bionic vision systems: i) resolution (<100 pixels) and recognition time (>1 s) limitations and ii) lack of long‐term durability and stability (under the atmosphere and repeat operation). In addition, multifunctional properties for health monitoring during blue‐ray surveillance should also be considered to avoid retinopathy under high‐energy visible light.

Optical sensing and synaptic devices have been integrated into artificial visual systems to realize recognition and memory functions for image preprocessing.^[^ ^11^, ^12^, ^13^, ^14^, ^15^ ^]^ Several novel technologies have been proposed to develop storage‐class memory and high reliability in visual systems, as listed in **Table** **1**,^[^ ^16^, ^17^, ^18^, ^19^, ^20^, ^21^ ^]^ including diode, resistive memory, and field‐effect transistor (FET) structures. The reported devices exhibited excellent optoelectronic properties at low driving voltages using vertical configurations but showed the characteristics of partial current crowding resulting in a relatively high current leakage, heat dissipation, and poor durability under long‐term operation. For example, Fan and co‐workers first fabricated an artificial eye using a hemispherical retina from a high‐density array of perovskite (PVSK) nanowires mimicking the photoreceptors in a human retina.^[^ ^16^ ^]^ Additionally, Zhou et al. developed a simple neuromorphic visual device based on two‐terminal optoelectronic resistive random access memory (ORRAM).^[^ ^17^ ^]^ The previous studies mostly focused on inorganic materials owing to their high electrical conductivity and carrier mobility for synaptic functions, such as PVSK,^[^ ^16^, ^22^, ^23^, ^24^ ^]^ transition metal dichalcogenides (TMDCs; MoS_2_ or MoO*_x_*),^[^ ^17^, ^19^, ^25^, ^26^ ^]^ carbon nitride (C_3_N_4_),^[^ ^20^ ^]^ PVSK/MoS_2_ hybrids,^[^ ^18^ ^]^ and *h*‐BN/WSe_2_ heterostructures.^[^ ^10^, ^27^ ^]^ However, the severe restrictions of a monotonic light response (limited UV range or monochromatic light only) and single function remain due to the narrow bandwidth of inorganic materials and complicated circuitry configurations. Unlike conventional photosensors, Liu and co‐workers demonstrated color differentiation between near‐infrared (NIR) and visible incidence with a preferred NIR selectivity via the combination of an organic photosensitive voltage divider and horizontal floating gate FETs (FGFETs), although the device was programmed under a relatively high driving voltage (−40 V) with a normal response time of 1000 ms.^[^ ^21^ ^]^ Thus, development of high‐performance multifunctional electronic devices is necessary to achieve a red/green/blue (RGB) photoresponse and an ultrafast image recognition rate for the next generation of smart artificial retinas of the human visual system.

**Table 1:** Comparison of photonic devices for retina applications with respect to the materials used, architecture, characteristics, and programming conditions

Device structure, active layer materials, and characteristics	Main restrictions	Driving voltage [V]	Response speed [ms]	Literature
–Diode –PVSK nanowire –Hemispherical array retina	Complicated circuitry design with different components	−3	19.2–23.9	Ref. ^[^ ^16^ ^]^
–ORRAM –MoO*_x_* –Simplified circuitry and reduced power consumption	Limited UV range	−4.5	<100	Ref. ^[^ ^17^ ^]^
–FETs –PVSK/MoS_2_ hybrid –Photosensory adaptation for red light response	High programming voltage	*V*_G_: −50; *V*_D_: 1	1000–3000	Ref. ^[^ ^18^ ^]^
–FETs –WSe_2_/BN heterostructure –Broadband spectrum distinction and multibit memory	High energy consumption	*V*_pro_: −80	50–3000	Ref. ^[^ ^19^ ^]^
–FETs –Carbon nitride –Photonic synapses and flexible	Limited UV range	*V*_G_: 0; *V*_D_: −5	20–2000	Ref. ^[^ ^20^ ^]^
–FGOFETs –Organic semiconductor (PIID, ROT300/VOPc, N1100) –Photo/electroresponsive, flexible	High programming voltage, complicated structure	−40	1000	Ref. ^[^ ^21^ ^]^
–OOSI –All organic semiconductors (DNTT‐ and PDI‐based materials) –Dual‐mode optical sensing for RGB photoreceptors and blue‐ray surveillance	–	±5	<250	This work

The abundant organic materials with unique features of long‐term biocompatibility, elasticity, and molecular diversity are promising candidates for flexible and bionic perception devices by incorporating organic semiconductors with charge transport and storage functions. This approach also provides tunable optoelectronic properties and facile‐controlled panel processing for further device scaling. Benefiting from the attractive advantages of i) simplified circuitry, ii) ultrafast signal transmission, iii) low energy consumption, and iv) wide bandwidth, and electrically/optically orthogonal operability, here, we propose an organic optical sensing inverter (OOSI) system with promising data storage for sensory memory and long‐term memory with a simultaneous light trigger. It enables image sensing and memory functions as well as neuromorphic visual preprocessing in the visual cortex of the human brain.

**Figure 1:**
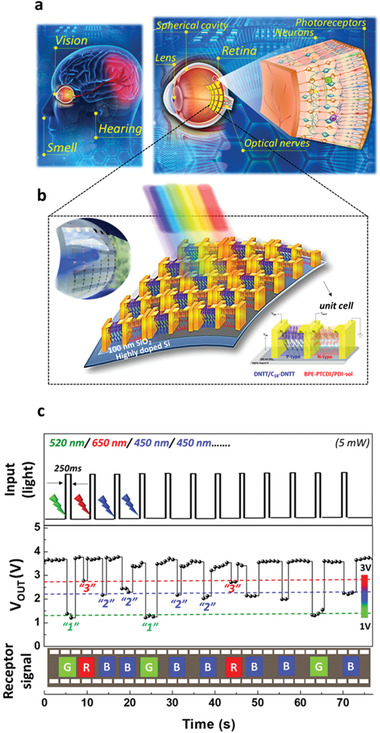
Design of a smart artificial retina based on an OOSI. a) Schematic of the human visual system. b) Crosspoint array architecture of the inverter device acting as retina‐like photoreceptors. c) Real‐time and light‐trigger‐dependent receptor signal.

The proposed smart artificial retina within a single cell using the OOSI device is shown in **Figure** **1** and consists of typical NMOS (N‐type metal oxide semiconductor field Effect transistor (MOSFET)) and PMOS (P‐type MOSFET) configurations. It acts not only as an RGB detector but also as a short‐wavelength (350–450 nm) recorder. To examine the optical‐switching function and real‐time preprocessing, we investigate the electrical characteristics of the light‐triggered phenomenon for both electron (N‐type)‐ and hole (P‐type)‐transporting devices. The light‐triggered characterizations show that our device exhibits high RGB responsivity and sensitivity, an ultrafast response speed (<300 ms) and a low energy consumption as low as ±5 V, which are suitable for wearable and bionic designs. The simulation of image recognition also demonstrates the basic function of the human eye in acquiring a 36 × 36 crosspoint array image (1296 pixels). More significantly, the proposed optical sensing inverter device is similar to the photoreceptors in the human retina and can thus potentially achieve reasonable image resolution and sensitivity for state‐of‐the‐art bionic systems. To the best of our knowledge, this is the first type of dual‐mode OOSI that can be operated both as RGB photoreceptors and for blue‐ray surveillance in a smart visual system.

## 2 Results and Discussion

### 2.1 Conception of the Artificial Retina Based on an OOSI

The human visual system has a pair of eyeballs for optical sensing, millions of nerve fibers for data transmission, and a brain for data processing. The internal structure of the human eye consists of a lens, a spherical cavity, and a hemispherical retina, which is the core component required to convert optical images to neuroelectric signals (Figure 1a). The schematic organization of the human retina includes ≈100–120 million photoreceptors as rod and cone cells, which play roles in low‐level light vision and color vision, respectively. A significant amount of the visual processing arises from the series of communications between neurons in the retina: i) At the transmitter release stage, the optical pulse is transformed into neuroelectric data throughout the connecting nerve tissue. ii) At an active termination stage, recognition images are built from the neuroelectric data by the optical nerves between the retina and visual cortex of the brain. In other words, this processing simultaneously combines two key components of optical sensing and memory functions of smart electronic devices, which will play a significant role in improving the quality of sensory data and further increasing the processing efficiency and accuracy, such as in image classification and recognition.

**Scheme 1:**
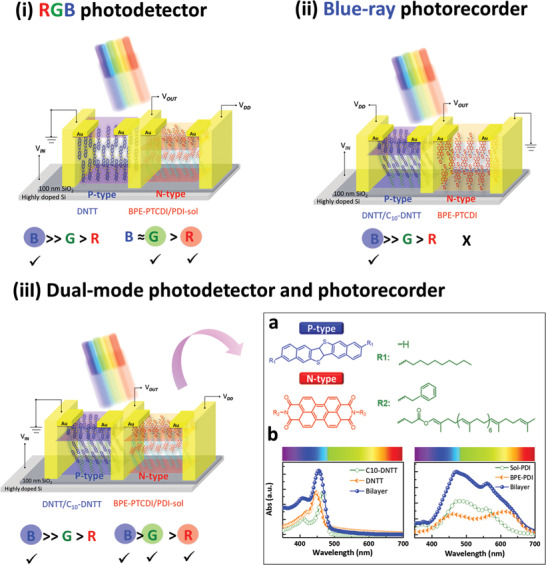
Design concept of a photoswitching inverter device based on organic materials and UV–vis absorption spectra of the studied molecular structures.

As shown in Figure 1b, a crosspoint array architecture can be designed based on the OOSI device, which has a time‐dependent output voltage that can act as the electrical signal of an artificial retina. In our recent work, the key factor of rod‐coil materials with conjugated core (rod) and long alkyl side chains (coils) can create charge‐trapping and tunneling, respectively.^[^ ^28^ ^]^ Perylene‐diimide (PDI)‐sol and C10‐DNTT (Dinaphtho[2,3‐b:2′,3′‐f]thieno[3,2‐b]thiophene) are used as dual functions of charge‐trapping (conjugated rod) and tunneling (insulating coil), while n‐type Bis(2‐phenyl‐ethyl) (BPE)‐PDI and p‐type DNTT are employed as the corresponding transporting layers. As shown in **Scheme** **1**, the absorption spectra for the two systems (N‐ and P‐channels) are clearly different in that the PDI‐based thin film possesses a broader absorption up to almost 700 nm, while the DNTT‐based thin film is only sensitive to a shorter wavelength, which will play roles in i) RGB sensory devices and ii) blue‐ray long‐term memory using electron‐ and hole‐controlled inverters with the single/bilayer systems, respectively. In order to significantly distinguish R, G, and B photoresponse in the N‐channel under light stimuli, the bilayer configuration of BPE‐PTCDI (perylene tetracarboxylic diimide)/PDI‐sol was used in both the transporting and memory layers for reducing the energy barrier in the interface and improving the RGB responsivity and sensitivity. Another purpose for the P‐ channel was the long‐term memory functionality to record the blue‐ray signal with the bilayer. On the contrary, the light‐switching electrical behavior exhibited the sensory memory based on the DNTT/BPE‐PTCDI single layer as shown in Figure S4 in the Supporting Information. Based on the above reasons, a dual‐mode function could be achieved via the combination of bilayer systems with a painstaking P–N‐type arrangement.

To confirm the broadband photoresponsive characteristics of the materials for color recognition, the OOSI device was subjected to random light illumination (blue, green, and red) with a pulse width of 250 ms under an input voltage (*V*_IN_) of nearly zero as shown in Figure 1c. Under light triggering, the corresponding values of the electrical signals (*V*_OUT_) are observed to significantly switch to multilevel states (defined by the numbers “3,” “2,” and “1”) according to light illumination at 650, 450, and 520 nm, respectively. Thus, the real‐time and light‐trigger‐dependent receptor signal can be quickly determined as R, G, and B colors for image classification. Note that the insert image of the OOSI device can also be fabricated on a flexible and transparent substrate, as shown in the insert image of Figure 1b.

### 2.2 Light‐Triggered Characteristics for Photodetectors

**Figure 2:**
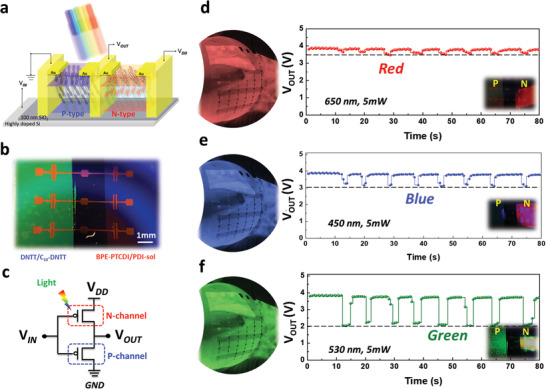
Light‐triggered electrical behavior. a) Device structure of the electron‐controlled inverter. b,c) Optical view of the inverter device and circuit configuration. d–f) Characterization of the time‐dependent voltage of the electron‐controlled inverter under R, B, and G stimuli (*V*_IN_ = 3.5 V; *V*_DD_ = 4 V).

The field‐programmable characteristics highly depend on the reconstruction of the complex routing matrix as the logic function of each individual building component and are thus practically less efficient.^[^ ^29^, ^30^, ^31^ ^]^ This is possibly a determining factor that could help simplify the architecture via programmable light‐triggered circuits integrated with sensor and long‐term memory functions in a single memlogic cell.^[^ ^32^, ^33^, ^34^ ^]^ However, a conventional complementary metal‐oxide semiconductor and resistive memory of image sensors of an artificial retina usually cause irreversible damage when repetitively accessing stored signal and readout information under long‐term light stimuli. Here, the fabricated OOSI device can be programmed by optical stimuli and output a real‐time voltage, which allows effective and expeditious conversion of these stimuli into receptor signals compared to a real‐time current for image processing. Thus, the use of this device is a strategy to overcome the resolution, responsive time, and long‐term durability limitations mentioned previously. The optical view and circuit configuration of the electron‐controlled inverter under light stimuli are shown in **Figure** **2**a–c. The characteristics of the typical inverter w/o the light‐triggered phenomenon are described in Note S1 and Figures S1 and S2 in the Supporting Information.

To explain the electrical characteristics of the light‐triggered phenomenon, we first examined the transfer curves based on P‐ and N‐MOSFET as shown in Figure S3b in the Supporting Information. When light illuminance at N‐channel, holes temporarily stored in the interfacial surface resulting in the shifting of threshold voltage and increment of current at *V*_g_ = 0. Thus, we could precisely define logic levels of “0” or “1” for the electrical signal. For the optical‐switching behavior of OOSI device, we measured the transfer curves of *V*_IN_ − *V*_OUT_ (*V*_IN_ from 0 to 5 V; *V*_DD_ of 5 V) with light stimuli. The intersection of the transfer curves obviously shifted to left according to various wavelengths (R, B, and G) as shown in Figure S5a in the Supporting Information. As a result, the real‐time characteristics are following examined and defined as levels of “R,” “B,” and “G” corresponding their light‐triggered phenomenon via fixed *V*_IN_ of 3.5 V. Thus, it indicates that tendency from a higher *V*_OUT_ to a lower *V*_OUT_ is observed in our proposed OOSI device. Particularly, in the case of the electron‐controlled inverter, the effects of electric competition under a low *V*_IN_ of 3.5 V, which plays an opposite role in erasing the stored charge at the same time, must be considered. On the other hand, the light‐responsive trend of the OOSI device with the light power densities of 1, 5, 10, and 20 mW is shown in Figure S5b in the Supporting Information. The switching *V*_OUT_ of the OOSI device to R, B, and G incident lights are 4.953.55, 3.422.44, and 2.841.55 V, respectively. 3D bars with light wavelength, powers intensity, and *V*_OUT_ signal are summarized in Figure S5c in the Supporting Information. For the gain properties of inverter, the peak of voltage gain of the N‐inverter, |d*V*_OUT_/d*V*_IN_|, is measured to be 2.89 at a supply voltage *V*_DD_ of 4 V w/o light stimuli (Figure S6, Supporting Information). Interestingly, it significantly enhances from 3.3 to 4.82 under light‐trigger despite the nonoptimal intersection of the transfer curves at the line of *V*_OUT_ = *V*_IN_. Notably, the real‐time output voltage in our device exhibits not only significant multilevel states for a random broadband (RGB) light detector but also an ultrafast response speed (<300 ms). Compared to other studies on optically dependent current devices, the ultrafast response based on the concept of the OOSI device highlights its great potential as the photosensor device of an artificial retina.^[^ ^18^, ^21^ ^]^

### 2.3 Nonvolatile Switching Characteristics for Blue‐Ray Recorders

**Figure 3:**
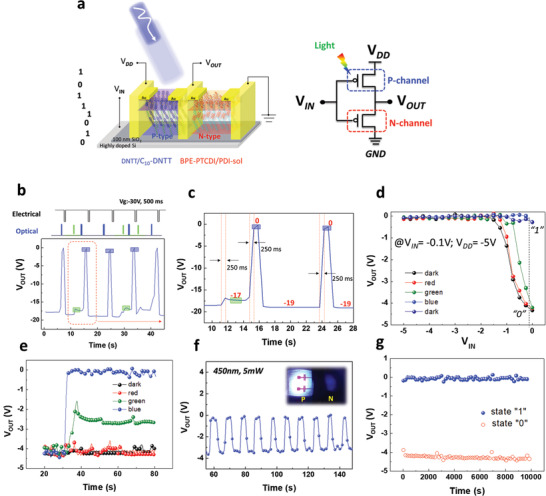
Nonvolatile switching characteristics. a) Hole‐controlled inverter (device and circuit configuration). b–e) Characteristics of the output voltage under R, B, and G. f,g) RWER cycles (read–write–erase–read) and retention time characteristics.

Light‐programmable photonic devices have attracted great interest due to over 80% of the information from an external environment entering the human vision under long‐term exposure to ultraviolet light. From this aspect, the degree of retinal damage is related to the intensity of the light source, the distance from the light source, and the exposure time. According to the suggested exposure limit of the International Commission on Nonionizing Radiation Protection, the spectral irradiance must be lower than 1 W m^−2^ (*t* > 100 s) for high‐energy visible light. Therefore, a multifunctional component within a single cell for health monitoring of artificial retinas during light surveillance must be developed to avoid retinopathy under high‐energy visible light, as shown in **Figure** **3**a.

Based on the above results of the electron‐controlled OOSI, the characteristics of the RGB sensing phenomenon for photodetectors are attributed to the effects of electric pulse competition with optical stimuli. Next, this leads us to consider the optical and electrical behavior of a hole‐controlled inverter based on a DNTT‐based thin film, which is only responsive to a shorter wavelength (blue rays) according to its UV–vis absorption spectrum (Scheme 1). Interestingly, the output voltage characteristics exhibit nonvolatile light‐switching behavior under blue/green/red stimuli with an intensity of 5 mW cm^−2^, especially for 450 nm (blue), with a dramatic transformation of *V*_OUT_ (Figure S8, Supporting Information). In addition, an ultrafast response (<250 ms) and a stable performance between “light‐on” and “electrical‐off” bistable states can be repetitively operated for random light‐stimuli real‐time sensing (Figure 3b,c).

Additionally, the unbalanced hole and electron carrier mobilities in the studied OOSI systems result in asymmetrical switching intersection of the transfer curves with a right‐shifting intersection. This suggests that optical‐switching behavior occur at nearly zero voltage. This phenomenon can logically become a design for power consumption (*V*_DD_) as low as −5 V with a readout voltage of −0.1 V (*V*_IN_), as shown in Figure 3d,e. Furthermore, our studied device also exhibits stable RWER (read–write–erase–read) cycles for 100 cycles and reliable long‐term stability over 10^4^ s (blue light‐on: *V*_OUT_ of 0 V; electrical‐off: *V*_OUT_ of −4 V) (Figure 3f,g and Figure S9, Supporting Information). Again, the dual‐mode design concept of the hole‐controlled component could broaden the horizons for smart artificial retinas in the future. Note that extra data a statistical analysis of the 36 devices’ performance (w/o or w/light stimuli by R/B/G and fabrication yield in four different batches) has been summarized in Figure S10 and Table S1 in the Supporting Information.

### 2.4 Simulation of Image Recognition with Preprocessing

**Figure 4:**
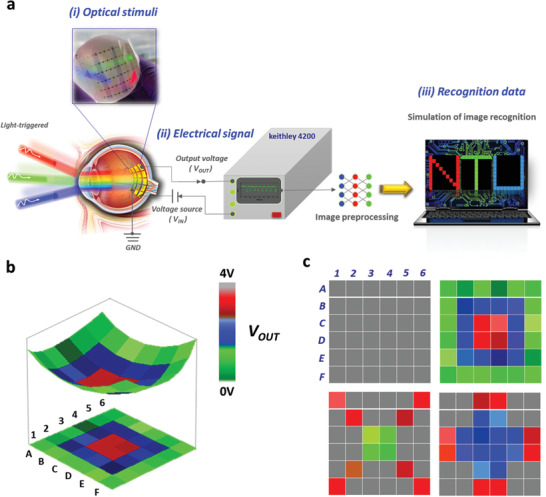
a) Schematics of the human visual system based on the OOSI and b,c) the simulation the image recognition of patters.

**Figure****4**a depicts a schematic of the human visual system: i) The visual information first stimulates and is detected by the fabricated OOSI device, acting as the artificial retina in a human eye; ii) the electric signal is transformed into the output voltage (*V*_OUT_), which is further combined and connected into the voltage detector and converter; and iii) then, the preprocessed image of color is transferred to a computer to simulate the image recognition function by ImitationEyes‐2.0, which is programming software for real‐time and random conditions. Detailed information on the experimental data and simulation is given in Note S2, Figure S8, and Tables S2–S4 in the Supporting Information, which demonstrate the color response schematic of the matrix after selective exposure, image preprocessing, and recognition. We have fabricated and measured point‐to‐point (6 × 6 pixels: A1, A2, A3,…) of the electrical signal (*V*_OUT_) under light stimuli through a crossarray shadow mask. Note that the exposed area dovetailed nicely with channel area of each cell. It suggests that the exposed individual three different pixel groups of “N,” “T,” and “U” can be recognized for R, G and B color, respectively. (Red: A1, A2, A3, A4, A5, A6, B2, C3, D4, E5, F1, F2, F3, F4, F5, F6; Green: A1, B2, C1, C2, C3, C4, C5, C6, D1, D2, D3, D4, D5, D6, E1, F1; Blue: A1, A2, A3, A4, A5, A6, B6, C6, D6, E6, F1, F2, F3, F4, F5, F6) In this case, we defined as level of 3.6–3.9, 2.8–3.4, and 1.8–2.6 V for “R,” “B,” and “G,” respectively. Thus, the image “NTU” is significantly captured in the matrix, in which “N”, “T,” and “U” are recorded in the R, G, and B light stimuli, respectively.

To further confirm the accuracy of the color sensing property of an individual pixel and recognition of a 6 × 6 crosspoint array image, three lasers with different light wavelengths (red, blue, and green) were used to trigger the selected pixel in sequence, and the light‐triggered output voltage (*V*_OUT_) was converted to a range of 0–4 V. Figure 4b shows that the hemispherical shape of the OOSI device ensures a more consistent distance between the pixels and the lens according to a 3D model. Note that this angle of view can be further simulated to approach the static vertical field of view of a human eye of ≈130°. Moreover, we also demonstrate three types of patterns with different RGB colors, as shown in Figure 4c, which suggests significant image recognition via the light‐triggered inverter device.

## 3 Conclusion

In summary, we developed a smart artificial retina via an OOSI that could be operated not only as RGB photoreceptors but also for blue‐ray surveillance in a visual system. The device exhibited a broadband light‐triggered response and a low energy consumption as low as ±5 V, overcoming the main restrictions for artificial retina application listed in Table 1. Notably, the real‐time output voltage showed significant multilevel states for a random broadband (RGB) light detector with an ultrafast response speed (<300 ms) compared to light‐dependent current sensory devices. Moreover, we also combined the multifunctional component within a single cell for health monitoring of an artificial retina during light surveillance to avoid retinopathy under high‐energy visible light. Our proposed OOSI device has great potential application in visual systems by simplifying the circuitry and providing dual‐mode functions, which could broaden the horizons for next‐generation smart artificial retinas.

## 4 Experimental Section

### Device Fabrication

Both N‐Type and P‐Type Bilayer Heterojunction FET Devices for NMOS and PMOS Configurations: A 100 nm SiO_2_ layer as a gate dielectric was thermally grown on highly N‐type doped Si (100) substrates. First, ≈40 nm of a Sol‐PDI thin film was spin coated on an octadecyltrichlorosilane (ODTS)‐modified substrate as the memory layer using a solution concentration of 4 mg mL^−1^ in chloroform. Then, the charge‐transporting layer of 40 nm BPE‐PDI was consequently deposited by a thermal evaporator (JIANFU, TE‐400) through a shadow mask for NMOS. Second, C10‐DNTT and DNTT thin films were prepared using a thermal evaporator with a deposition rate of 0.3–0.5 Å s^−1^ through a shadow mask for the P‐type FET of the inverter device. Finally, 60 nm contact gold electrodes were deposited through a crossarray shadow mask with channel width (*W*) and length (*L*) defined as 50 and 1000 µm, respectively.

### Characterizations

For the electrical characterizations, the entire measurement processes were conducted in a dark environment using a Keithley 4200‐SCS semiconductor parameter analyzer (Keithley Instrument Inc., Cleveland, OH, USA) in a N_2_‐filled glove box. Various light illumination was performed with different light sources (450 nm laser with an intensity of 5 mW cm^−2^, 530 nm laser with an intensity of 5 mW cm^−2^, and 650 nm laser with an intensity of 5 mW cm^−2^). The intensity of the laser source was adjusted by using an optical attenuator (neutral density filter) and was calibrated with a laser power meter (Thorlabs PM 100D). For visualizing the film morphologies and optical characterization, all the prepared films were deposited onto an ODTS‐modified silicon wafer or cleaned quartz depending on the experimental demand.

## Conflict of Interest

The authors declare no conflict of interest.

## Author Contribution

C.‐C.H. and Y.‐C.C. contributed equally to this work. C.‐C.H., Y.‐C.C., and W.‐C.C. conceived and designed the study. W.‐C.C. supervised the project. C.‐C.H. and Y.‐C.C. performed the experiments, including both fabrication and characterization. C.‐C.H., Y.‐C.C., and Y.‐C.L. performed the simulations. C.‐C.H., Y.‐C.C., and W.‐C.C. cowrote the paper. All the authors discussed the results and commented on the paper.

## Supporting information

Supporting Information

## Data Availability

Research data are not shared.
