# Supplementary material for: Conception of a Smart Artificial Retina Based on a Dual‐Mode Organic Sensing Inverter
Source: Adv Sci (Weinh). 2021 Jun 6;8(16):2100742. doi: 10.1002/advs.202100742 (PMC8373107; doi:10.1002/advs.202100742)
Supplement: Supplementary file 1 — Supporting Information [file ADVS-8-2100742-s001.pdf]

© 2021 Wiley-VCH GmbH

ADVANCED SCIENCE Open Access

Supporting Information

for *Adv. Sci.*, DOI: 10.1002/advs.202100742

Conception of a Smart Artificial Retina Based ona Dual-mode Organic Sensing Inverter

*Chih-Chien Hung, Yun-Chi Chiang, Yan-Cheng Lin, Yu-Cheng Chiu, and Wen-Chang Chen**

Supporting Information

Conception of a Smart Artificial Retina Based on a Dual-mode Organic Sensing Inverter

Chih-Chien Hung,[a,b] *Yun-Chi Chiang,*[b] *Yan-Cheng Lin,*[a,b] *Yu-Cheng Chiu* [b,c] *and Wen-Chang Chen* [a,b,]*

[a]Dr. C.-C. Hung, Dr. Y.-C. Chiang, Dr. Y.-C. Lin, and Prof. W.-C. Chen
Department of Chemical Engineering, National Taiwan University, Taipei 10617, Taiwan

[b]Dr. C. C. Hung, Dr. Y.-C. Lin, Prof. Y.-C. Chiu, and Prof. W.-C. Chen
Advanced Research Center for Green Materials Science and Technology, National Taiwan University, Taipei 10617, Taiwan.

[c]Prof. Y.-C. Chiu
Department of Chemical Engineering, National Taiwan University of Science and Technology,
Taipei 10607, Taiwan.

*Corresponding authors. email:
chenwc@ntu.edu.tw (W.-C. Chen)

Supplementary Note 1

Electrical characteristics of typical inverter

These typical inverter devices consist of DNTT/C_{10}-DNTT and BPE-PTCDI/PDI-sol for P-channel and N-channel, respectively**.** The circuit configurations for hole- and electron-controlled inverter devices as shown in **Supplementary Fig. 1a.** The input voltage (V_{IN}) was applied to the common Si back gate. The supply voltage (V_{DD}) was connected to the source electrode of the DNTT-based and PDI-based field-effect transistors (FET) for the hole-controlled and electron-controlled inverter, respectively and the source electrode (Au) in the device was grounded (*GND*). The output voltage (V_{OUT}) according to the input voltage was measured in the middle drain electrode, which is shared by the P-channel FET and N-channel FET.

Depending on the polarity of the supply drain voltage (V_{DD}), one can observe well-defined voltage-transfer characteristics in the first and third quadrant of the output versus

input voltage diagram (±5 to ±40). We note that the transfer characteristics show asymmetrical switching because of unbalanced electron and hole mobilities, as graphically shown in **supplementary Fig. 1b** (the ideal intersection of the transfer curves at the line of $V_{OUT}=V_{IN}$). Despite this, the asymmetrical phenomenon is a key factor between voltage-driven and photo-trigger competition in OOSI, thus the effects of w/o light-triggered phenomenon will be logically investigated on the both N-type and P-type transporting systems for those electrical behaviors in the forthcoming sections. Note that these typical transfer and output curves of DNTT- and BPE-PTCDI-based FETs as shown in **supplementary Fig 2**. The FETs from this particular circuit showed current ratios (I_{on}/I_{off}) greater than 10^5 and the saturation hole and electron mobilities were 0.84 and 1.43×10^{-2} cm^2/Vs for P-channel and N-channel, respectively.

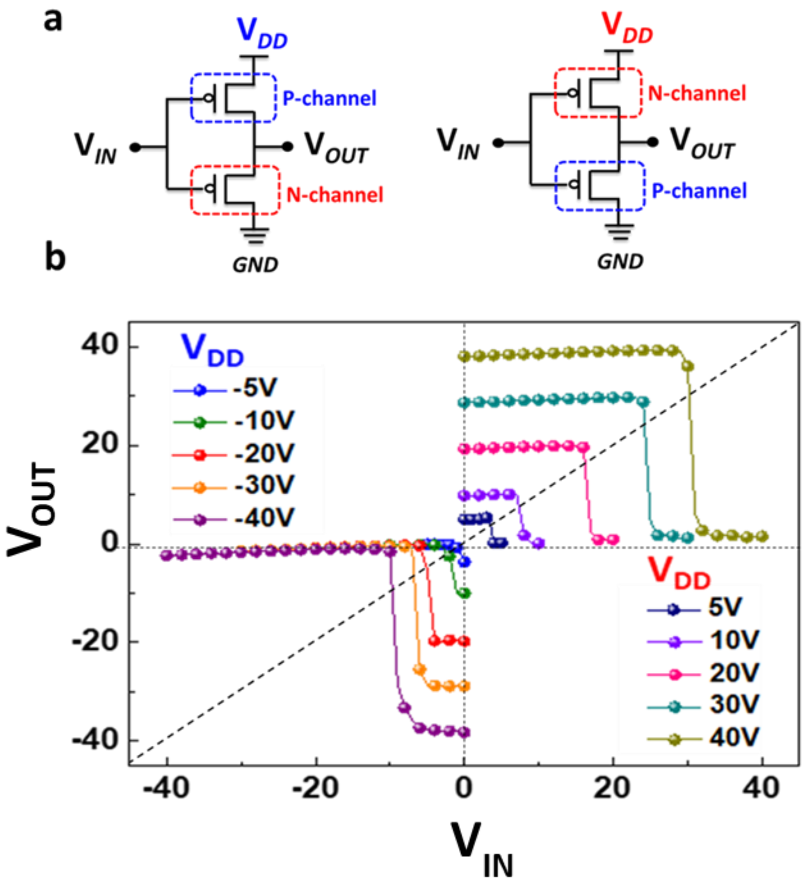

Supplementary Fig. 1. | Electrical characteristics of OOSID. **a**, Circuit configurations for hole- and electron-controlled inverter devices. **b**, Voltage-transfer characteristics of the output versus input voltage diagram (±5 to ±40).

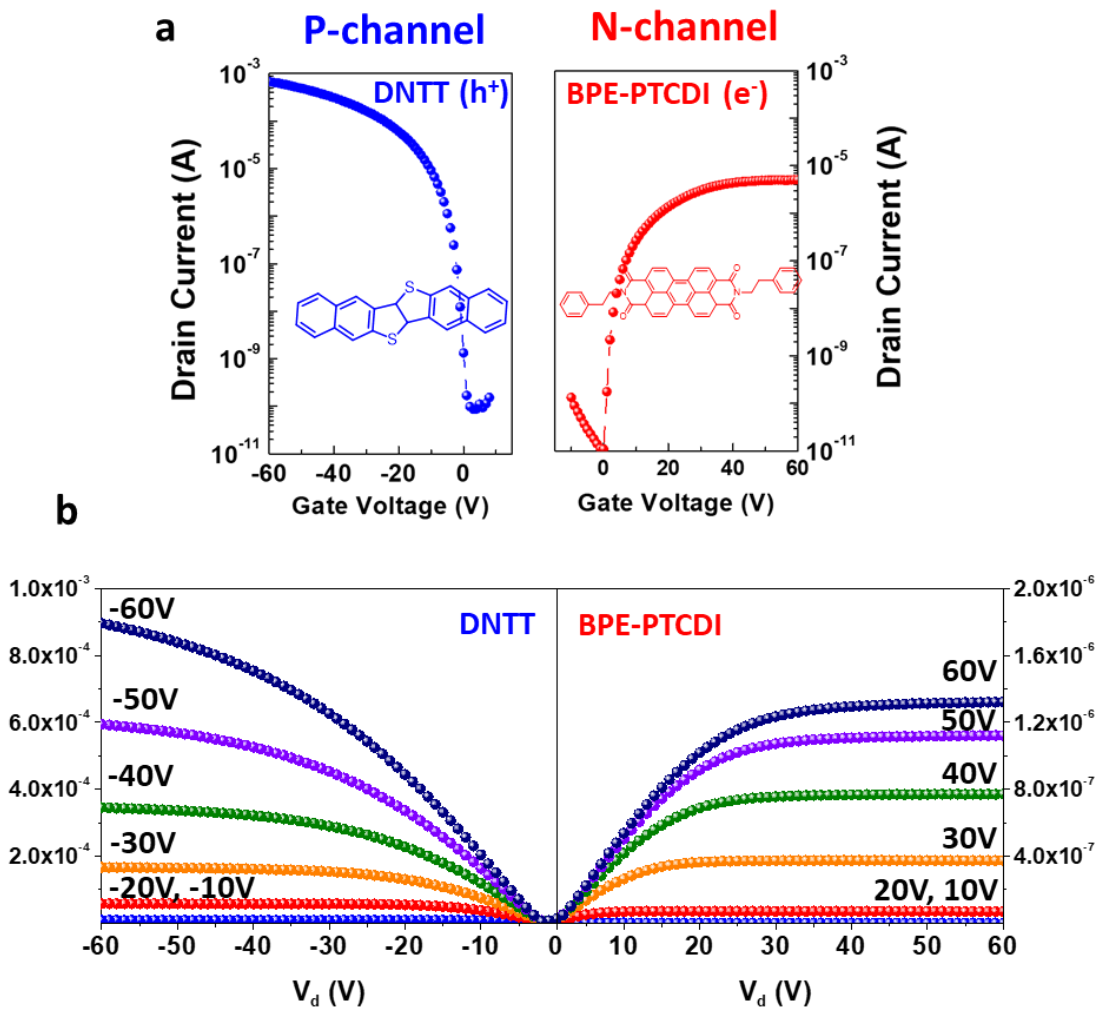

Supplementary Fig. 2. | Typical transfer and output curves of P- and N- channel FETs.

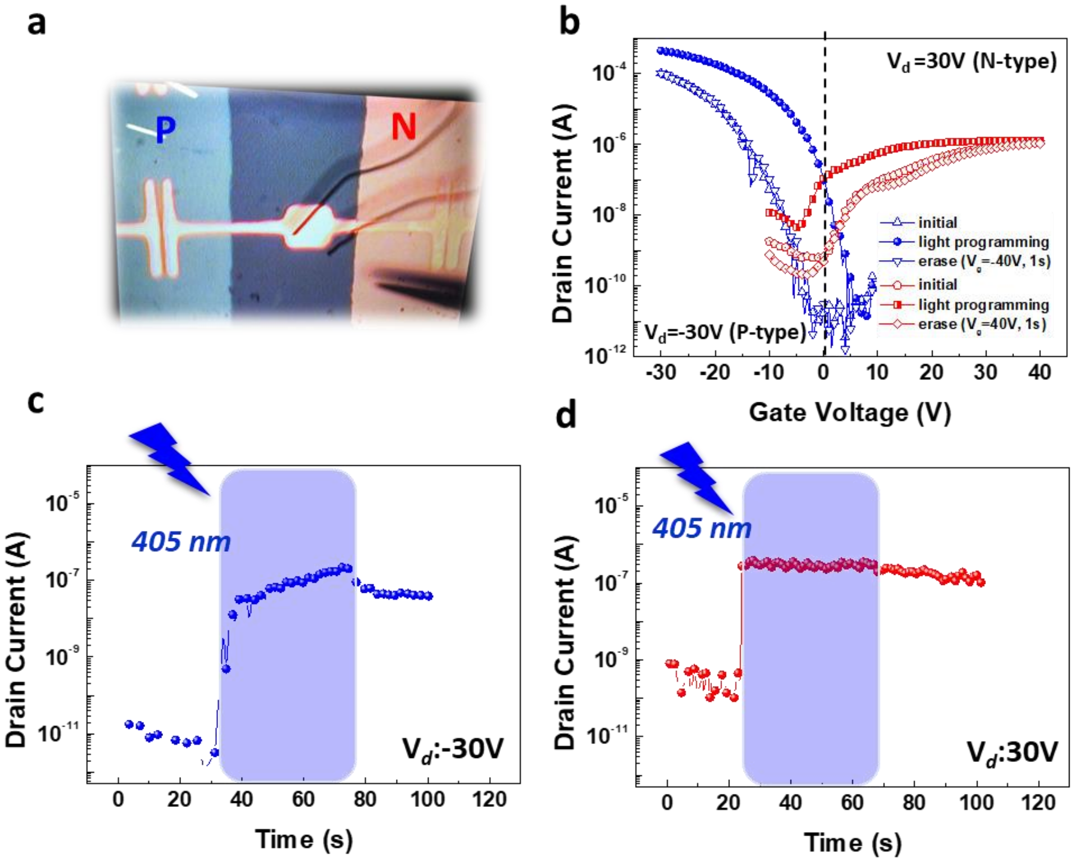

Supplementary Fig. 3. | a, Optical view of inverter device consist of typical NMOS (N-type MOSFET) and PMOS (P-type MOSFET) configurations. **b,** Photoprogramming characteristic of transfer curves based on PMOS and NMOS. **c-d**, temporal I_{sd} curves using different light sources for P- and N-channel FETs devices. Note that devices were operated at V_d= -30V or 30V with V_g=0.

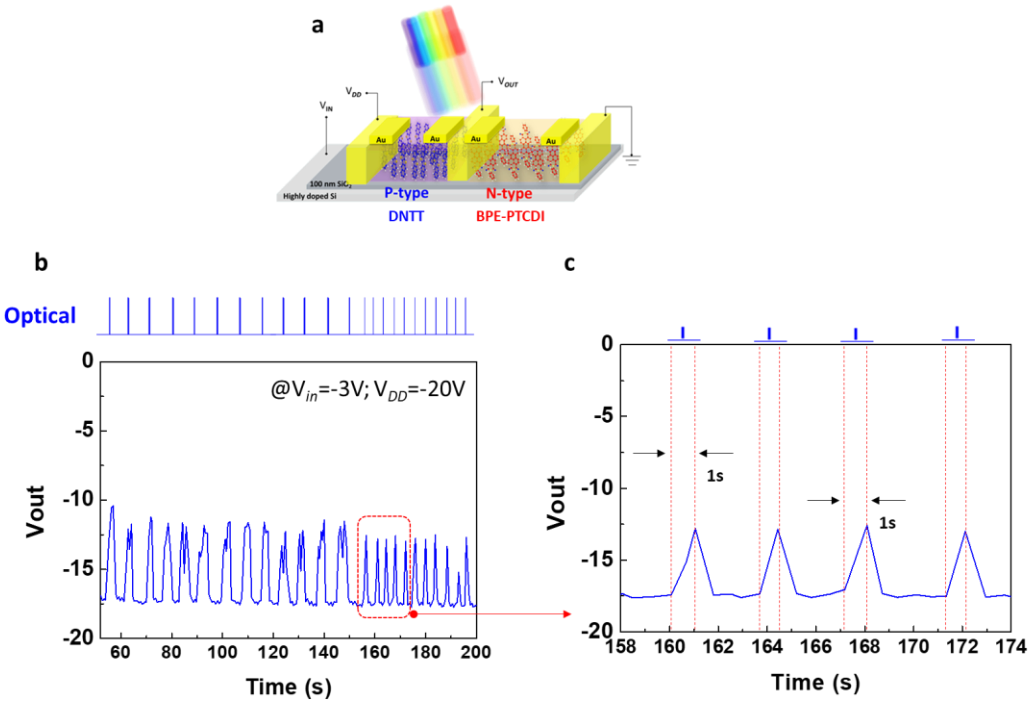

Supplementary Fig. 4. | Light-switching electrical behaviors based on DNTT/BPE-PTCDI single layer inverter. a, device configuration. **b-c,** Photoprogramming characteristic of the output voltage.

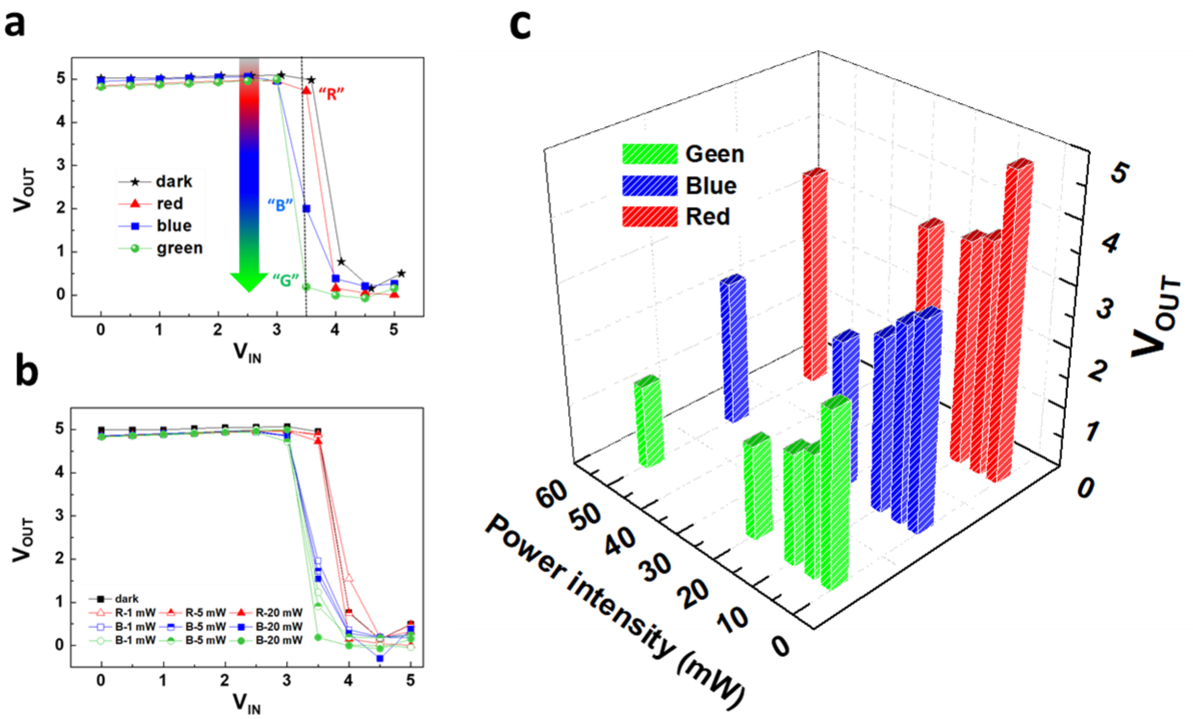

Supplementary Fig. 5. | a-c. The characterization of curve (V_{IN}-V_{OUT}) and 3D Bars with different light powers of RGB.

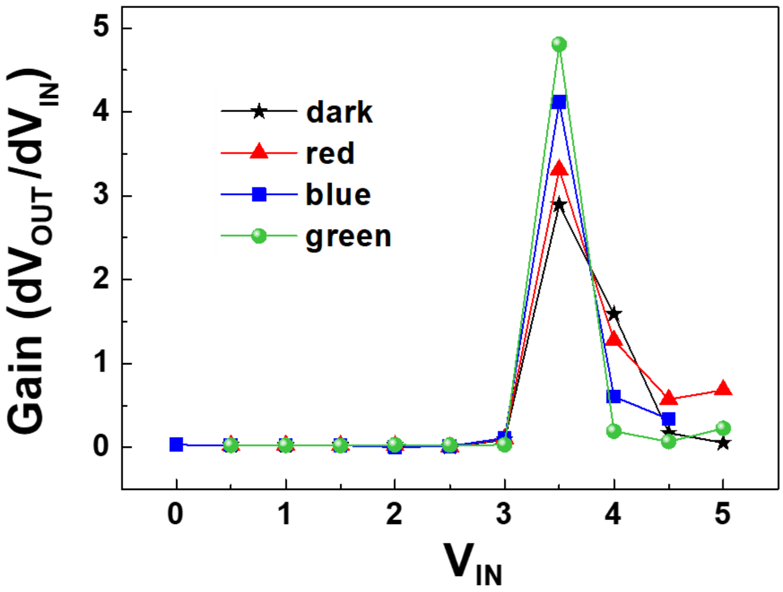

Supplementary Fig. 6. | The peak of voltage gain based on electron-controlled inverter w/o and with light stimuli of R, B and G, respectively.

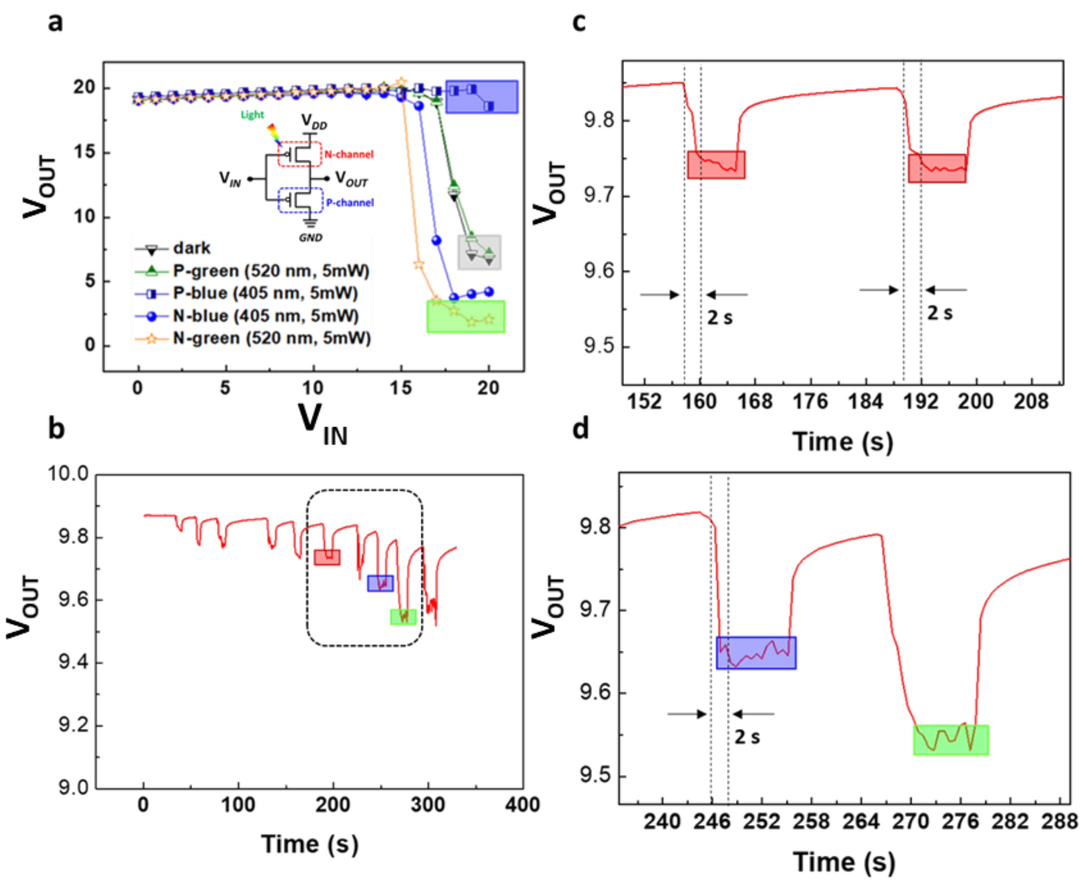

Supplementary Fig. 7. | a, Photoprogramming characteristic of the output voltage for electron-controlled inverter. **b-d**, The real-time and light-triggered-dependent voltage.

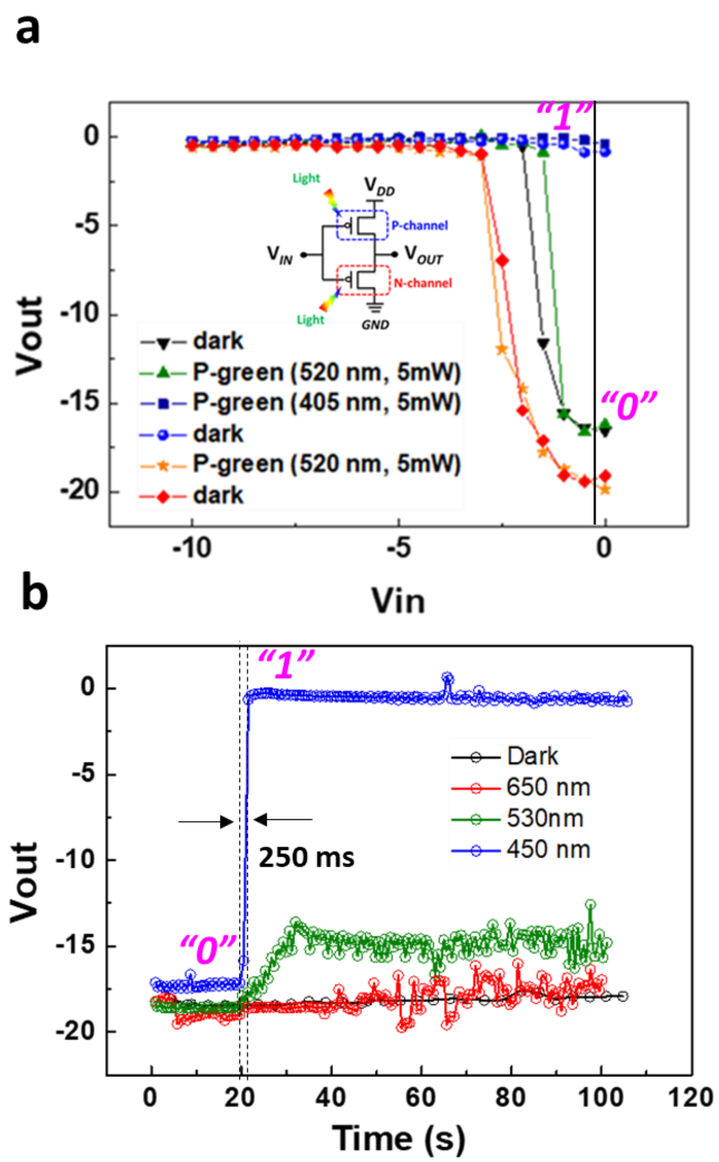

Supplementary Fig. 8. | a-b, Photoprogramming characteristic of the output voltage for hole-controlled inverter.

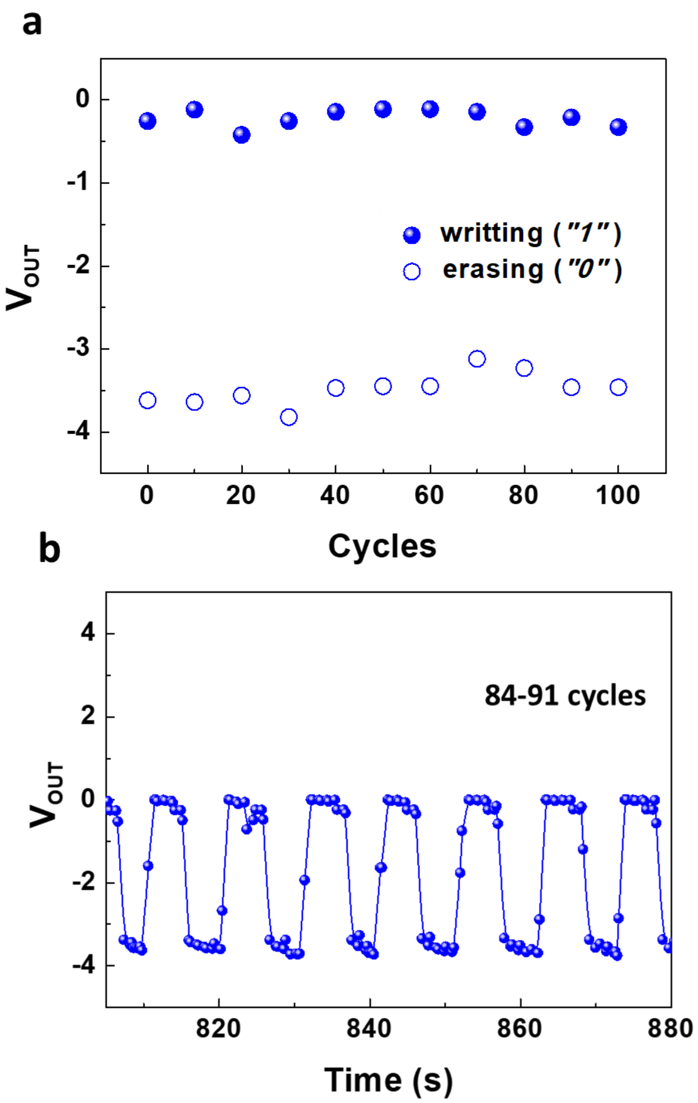

Supplementary Fig. 9. | a. Cyclic endurance on OOSI device for 100 cycles. **b.** The Characterization of 84-91 cycles.

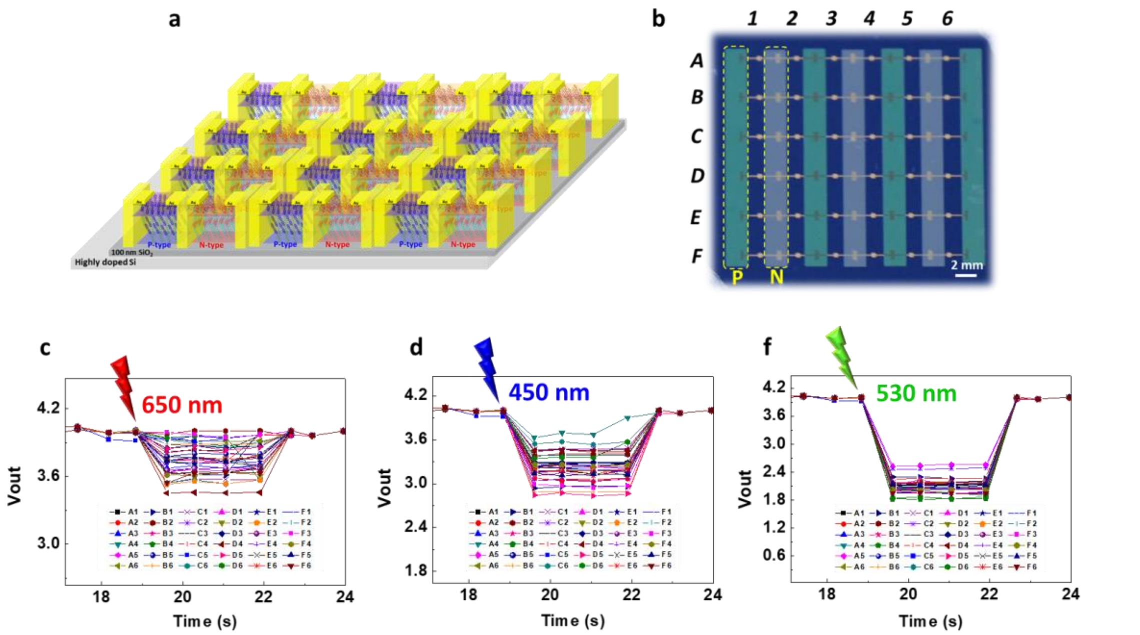

Supplementary Fig. 10. | a, Schematic diagrams of the fabricated OOSI and **b**, Optical view of the 6×6 cross-point array. **c-f**, Characterization of 36 devices for the electron-controlled inverter under R, B and G stimuli.

Supplementary Note 2

The simulation the image recognition of patters.

Supplementary Fig. 11 shows the 3 examples of the images with single-color digits and mixed-color images, which are re randomly generated by ImitationEyes-2.0 by $c^{\#}$.net for the 36 pixels (A1、A2、A3…, et al) to trigger the selected pixel in the matrix. First, we defined multilevel states of 2.0 ± 0.8, 3.0 ± 0.5 and 3.6 ± 0.3 as G, B and R color by 36 cells of OOSI device. Next, those individual single-color pixels of 6×6 cross-point array image are listed in **Supplementary table 2-4.** for 3 type of patterns, and finally corresponding output voltages (V_{OUT}) then convert them into a recognition output image (type1-type3). Note that a light triggered V_{OUT} is converted to a range of 0 to 4 V under blue/green/red (G/B/R) sitimuli with an intensity of 5 m Wcm^{-2}.

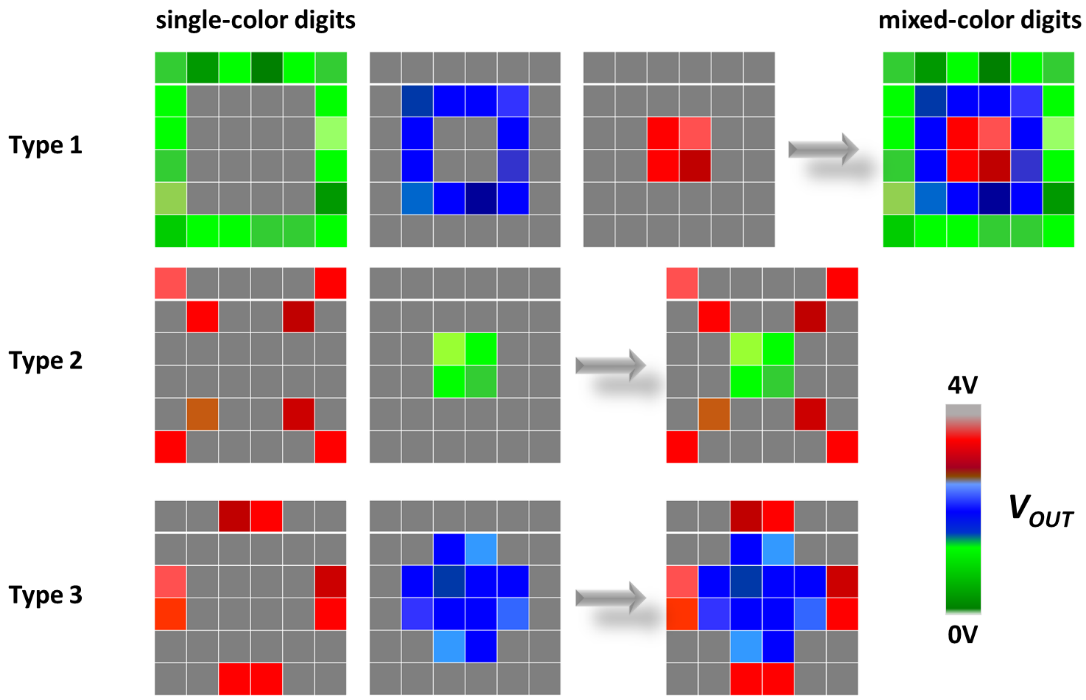

Supplementary Fig. 11. | The simulation the image recognition. 3 type of patterns with different RGB single-color digits and their mixed-color digits.

Supplementary Table 1. | The electrical performance was averaged from at least 36 devices in four different batches.

State of devices	w/o light stimuli (V_{OUT}) [a]	w/ light stimuli (V_{OUT}) [a]	Yield(%)
Red -detector	4.0±0.04	3.7±0.17	72.2
Blue-detector		3.2±0.18	77.8
Green-detector		2.0±0.13	83.3
Blue-recorder	-3.6±0.06	-0.1±0.04	91.7

[a] Note that all devices were measured at V_{DD} of 4 V; V_{IN} of 3.5 V and V_{DD} of -5 V; V_{IN} of -0.1 V for photodetector and photorecorder, respectively.

Supplementary Table 2. | The simulation the image recognition of 36 pixels for type 1 mixed-pattern.

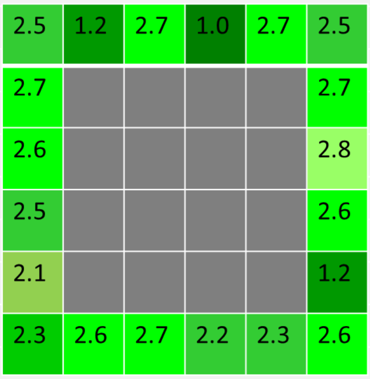

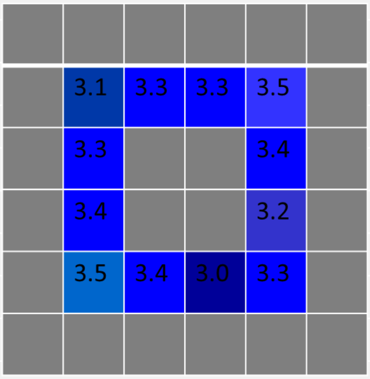

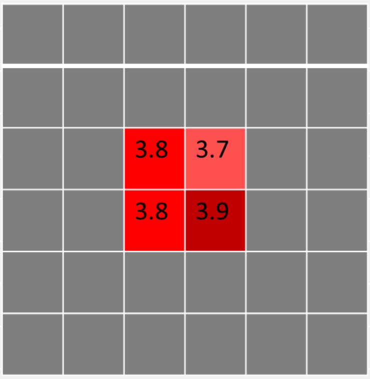

Single-color digits	Pixel No.	G	B	R
	A1	2.5	4.0	4.0
	A2	1.2	4.0	4.0
	A3	2.7	4.0	4.0
	A4	1.0	4.0	4.0
	A5	2.7	4.0	4.0
	A6	2.5	4.0	4.0
	B1	2.7	4.0	4.0
	B2	4.0	3.1	4.0
	B3	4.0	3.3	4.0
	B4	4.0	3.3	4.0
	B5	4.0	3.5	4.0
	B6	2.7	4.0	4.0
	C1	2.6	4.0	4.0
	C2	4.0	3.3	4.0
	C3	4.0	4.0	3.8
	C4	4.0	4.0	3.7
	C5	4.0	3.4	4.0
	C6	2.8	4.0	4.0
	D1	2.5	4.0	4.0
	D2	4.0	3.4	4.0
	D3	4.0	4.0	3.8
	D4	4.0	4.0	3.9
	D5	4.0	3.2	4.0
	D6	2.6	4.0	4.0
	E1	2.1	4.0	4.0
	E2	4.0	3.5	4.0
	E3	4.0	3.4	4.0
	E4	4.0	3.0	4.0
	E5	4.0	3.3	4.0
	E6	1.2	4.0	4.0
	F1	2.3	4.0	4.0
	F2	2.6	4.0	4.0
	F3	2.7	4.0	4.0
	F4	2.2	4.0	4.0
	F5	2.3	4.0	4.0
	F6	2.6	4.0	4.0

Supplementary Table 3. | The simulation the image recognition of 36 pixels for type 2 mixed-pattern.

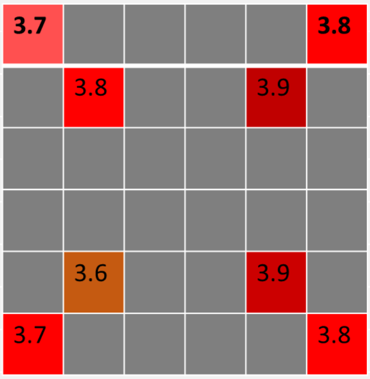

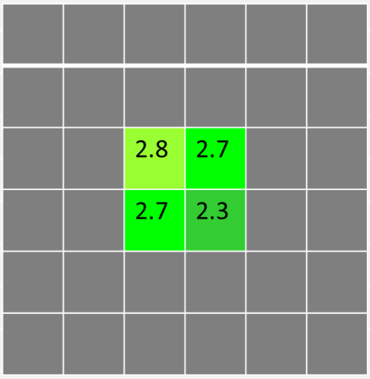

Single-color digits	Pixel No.	G	B	R
	A1	4.0	4.0	3.7
	A2	4.0	4.0	4.0
	A3	4.0	4.0	4.0
	A4	4.0	4.0	4.0
	A5	4.0	4.0	4.0
	A6	4.0	4.0	3.8
	B1	4.0	4.0	4.0
	B2	4.0	4.0	3.8
	B3	4.0	4.0	4.0
	B4	4.0	4.0	4.0
	B5	4.0	4.0	3.9
	B6	4.0	4.0	4.0
	C1	4.0	4.0	4.0
	C2	4.0	4.0	4.0
	C3	2.8	4.0	3.8
	C4	2.7	4.0	3.7
	C5	4.0	4.0	4.0
	C6	4.0	4.0	4.0
	D1	4.0	4.0	4.0
	D2	4.0	4.0	4.0
	D3	2.7	4.0	3.8
	D4	2.3	4.0	3.9
	D5	4.0	4.0	4.0
	D6	4.0	4.0	4.0
	E1	4.0	4.0	4.0
	E2	4.0	4.0	3.6
	E3	4.0	4.0	4.0
	E4	4.0	4.0	4.0
	E5	4.0	4.0	3.9
	E6	4.0	4.0	4.0
	F1	4.0	4.0	3.7
	F2	4.0	4.0	4.0
	F3	4.0	4.0	4.0
	F4	4.0	4.0	4.0
	F5	4.0	4.0	4.0
	F6	4.0	4.0	3.8

Supplementary Table 4. | The simulation the image recognition of 36 pixels for type 3 mixed-pattern.

Single-color digits	Pixel No.	G	B	R
	A1	4.0	4.0	4.0
	A2	4.0	4.0	4.0
	A3	4.0	4.0	3.9
	A4	4.0	4.0	3.7
	A5	4.0	4.0	4.0
	A6	4.0	4.0	4.0
	B1	4.0	4.0	4.0
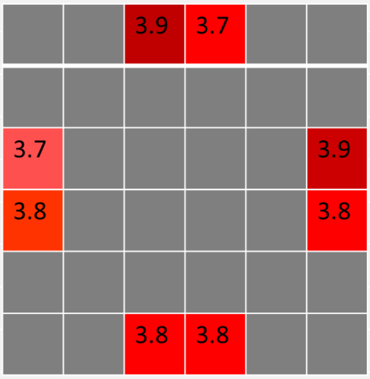	B2	4.0	4.0	4.0
	B3	4.0	3.3	4.0
	B4	4.0	3.5	4.0
	B5	4.0	4.0	4.0
	B6	4.0	4.0	4.0
	C1	4.0	4.0	3.7
	C2	4.0	3.3	4.0
	C3	4.0	3.1	4.0
	C4	4.0	3.3	4.0
	C5	4.0	3.4	4.0
	C6	4.0	4.0	3.9
	D1	4.0	3.5	3.8
	D2	4.0	3.3	4.0
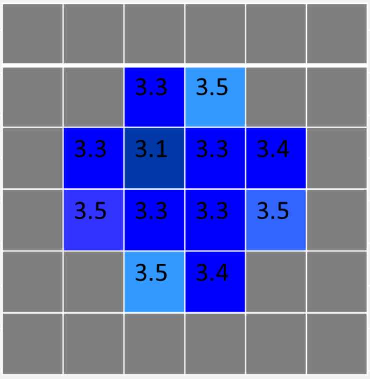	D3	4.0	3.3	4.0
	D4	4.0	3.5	4.0
	D5	4.0	4.0	4.0
	D6	4.0	4.0	3.8
	E1	4.0	4.0	4.0
	E2	4.0	4.0	4.0
	E3	4.0	3.5	4.0
	E4	4.0	3.4	4.0
	E5	4.0	4.0	4.0
	E6	4.0	4.0	4.0
	F1	4.0	4.0	4.0
	F2	4.0	4.0	4.0
	F3	4.0	4.0	3.8
	F4	4.0	4.0	3.8
	F5	4.0	4.0	4.0
	F6	4.0	4.0	4.0
